# Is Race Associated with the Surgical Treatment for Benign Prostatic Hyperplasia? An Analysis of 30,000 Medicare Lives

**DOI:** 10.1007/s40615-023-01538-0

**Published:** 2023-04-24

**Authors:** Gopal L. Narang, Sirikan Rojanasarot, Ben Cutone, Mitchell R. Humphreys

**Affiliations:** 1 University of North Carolina, Chapel Hill, NC USA; 2 Boston Scientific, Marlborough, MA USA; 3 Mayo Clinic, Scottsdale, AZ USA

**Keywords:** Benign prostatic hyperplasia, Racial disparities, Surgical treatment, Outcomes research

## Abstract

**Backgrounds:**

With an increased prevalence and burden of benign prostatic hyperplasia (BPH), effective and equitable treatment is a priority. Limited data exist evaluating treatment disparities for patients with BPH by race. This study examined the association between race and BPH surgical treatment rates among Medicare beneficiaries.

**Methods:**

Medicare claims data were used to identify men newly diagnosed with BPH from January 1, 2010 through December 31, 2018. Patients were followed until their first BPH surgery, a diagnosis of prostate/bladder cancer, termination of Medicare enrollment, death, or end of study. Cox proportional hazards regression compared the likelihood of BPH surgery between men of different races (White vs. Black, Indigenous, and People of Color (BIPOC)), controlling for patients’ geographical region, Charlson comorbidity score, and baseline comorbidities.

**Results:**

The study included 31,699 patients (13.7% BIPOC). BIPOC men had significantly lower BPH surgery rates (9.5% BIPOC vs. 13.4% White; *p*=0.02). BIPOC race was associated with a 19% lower likelihood of receiving BPH surgery than White race (HR, 0.81; 95% CI 0.70, 0.94). Transurethral resection of the prostate was the most common surgery for both groups (49.4% Whites vs. 56.8% BIPOC; *p*=0.052). A higher proportion of BIPOC men underwent procedures in inpatient settings compared to White men (18.2% vs. 9.8%; *p*<0.001).

**Conclusions:**

Among a cohort of Medicare beneficiaries with BPH, there were notable treatment disparities by race. BIPOC men had lower rates of surgery than White men and were more likely to undergo procedures in the inpatient setting. Improving patient access to outpatient BPH surgical procedures may help address treatment disparities.

**Supplementary Information:**

The online version contains supplementary material available at 10.1007/s40615-023-01538-0.

## Introduction

Benign prostatic hyperplasia (BPH) is one of the most prevalent chronic conditions affecting older men [1]. In the United States (US), BPH affects a majority of older men, especially those of the Medicare eligibility age [2]. BPH is associated with progressive lower urinary tract symptoms (LUTS) and bladder, urinary tract, or kidney problems [3]. Untreated BPH may result in serious complications including urinary retention (acute and chronic), hematuria, urinary tract infection, bladder stones, bladder wall damage, renal dysfunction, incontinence, and erectile dysfunction (ED) [4, 5]. BPH significantly impacts patients’ quality of life and poses a significant economic burden to the healthcare system [6].

As the global impact of BPH increases [7], the need for effective treatment options is increasing as well. The goals of BPH treatment are to improve bothersome LUTS and to prevent disease progression that would diminish health and result in invasive surgical procedures. With the development of minimally invasive surgical therapies, advancement of holmium laser enucleation of the prostate (HoLEP), and strides made in robotic surgery, there are numerous treatment options for urologists to employ in the treatment of men with BPH. It is important all treatment options are available to all BPH patients. Recent studies have identified racial and socioeconomic disparities in the treatment of BPH, with Black, Indigenous, and People of Color (BIPOC) patients shown to be undertreated relative to White patients [8–12]. Furthermore, where patients access care, including the service location for BPH surgeries, impacts treatment options.

This research addresses these specific gaps by evaluating the surgical rates of BPH treatment among a large cohort of newly diagnosed Medicare beneficiaries with BPH, paying particular attention to treatment rates by race. This research hypothesized that BIPOC men are less likely to receive surgical treatment for BPH than White men. Additionally, this study sought to examine patient factors associated with time to, type of, and site of BPH surgical procedure among Medicare enrollees with newly diagnosed BPH.

## Materials/Subjects and Methods

### Study Design

This retrospective cohort study used real-world patient-level data from the Medicare 5% Standard Analytical Files (SAF) from 2009 through 2019. The SAF contains beneficiary and fee-for-service administrative claims data. These data were retrospectively collected and de-identified; therefore, neither institutional review board approval nor informed consent was obtained.

All men newly diagnosed with BPH between January 1, 2010 and December 31, 2018, were identified. Study participants were followed from their earliest BPH diagnosis until their earliest claim date for (a) BPH surgery, (b) prostate or bladder cancer diagnosis, (c) the end of their continuous enrollment on a Medicare plan, (d) death, or (e) the end of the study period (December 31, 2019).

### Study Population

All male Medicare beneficiaries who had at least one BPH diagnosis claim and were continuously enrolled in the Medicare program for at least 1 year before the  earliest BPH diagnosis were eligible for this study. The presence of a BPH diagnosis was determined using the International Classification of Diseases, Ninth and Tenth Revisions, Clinical Modification (ICD-9-CM, ICD-10-CM) codes. Each subject’s index BPH diagnosis date was defined as the date of the earliest BPH diagnosis within the patient identification period. BPH diagnosis and procedure codes used for participant inclusion are included as Appendix 1.

Eligible study subjects were required to be 66 years of age during their index year, which ensured all subjects had a full year of Medicare claims data prior to index BPH diagnosis date, given their enrollment in Medicare at age 65. Furthermore, this requirement reduced the likelihood that each subject’s index BPH diagnosis was an incident case to provide a sample of comparable BPH severity. Given the differences in healthcare systems in the US territories, only men with BPH from the 50 states were included in the final analysis [13].

Study participants were divided by race (either White or BIPOC) using beneficiary race data. BIPOC included subjects in the following race categories: Black, Hispanic, Asian, North American Native, and Other.

### Study Outcomes

A key outcome for this analysis was a type of BPH surgery. BPH surgical events were categorized as either minimally invasive surgery (laser coagulation, PUL, TUIP, TUMT, TUNA, or WVTT) or invasive surgery (HoLEP, open simple prostatectomy, PVP/HoLAP, or TURP).

Patient characteristics included the geographical region of residence (South, Midwest, Northeast, or West) at the time of index BPH diagnosis. A Charlson comorbidity score 1 year prior to the  index BPH diagnosis was calculated for each study subject using the methods of Quan et al. (2005) updated weights from Quan et al. (2011) [14, 15]. The presence of six clinically relevant comorbidities (cardiovascular disease, diabetes mellitus, dyslipidemia, erectile dysfunction, hypertension, and obesity) within 1 year prior to the index BPH diagnosis date was determined using ICD-9-CM and ICD-10-CM codes.

### Statistical Analysis

Baseline patient characteristics were reported as counts and percentages for categorical variables. The distribution of covariates between White and BIPOC men was compared using the absolute average standardized difference (ASD) statistic (the difference in proportions between groups divided by the pooled standard deviation). A negligible group difference was defined as an ASD of less than 0.1 [16]. Kaplan-Meier statistics were used to compare the risk of BPH surgery for White versus BIPOC men. Cox proportional hazards regression models compared the likelihood of BPH surgery by race, controlling for patients’ geographical region of residence, Charlson comorbidity score, and baseline comorbidities. The hazard ratio (HR), 95% confidence interval, and the corresponding *p*-values were reported. Two-sided *p*-values of less than 0.05 were considered statistically significant. Data were analyzed using SAS 9.4 software (SAS Institute Inc. Cary, NC, USA).

## Results

### Patient Characteristics

**Fig. 1:**
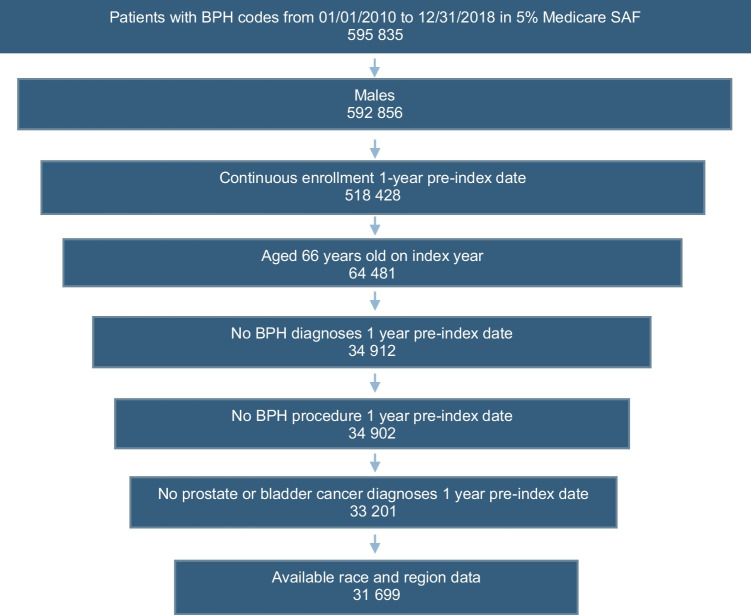
Flowchart of the selection of study populations

In this study, 31,699 male Medicare beneficiaries met the inclusion criteria; 86.3% were White, and 13.7% were BIPOC (Fig. 1). Nearly 40% of study participants had a Charlson comorbidity score of 0. The two most prevalent comorbid conditions were hypertension (70.9%) and dyslipidemia (66.9%). The geographic region of residence with the highest percentage of study participants was the South (40.6%). The median follow-up was 3.6 years (IQR 1.7–6.0) for White men and 2.8 years (IQR 1.3–5.2) for BIPOC men. The ASD values for these demographic and clinical attributes were <0.1, indicating comparable patient characteristics of the groups. Demographic and clinical characteristics of the study population are presented in Table 1.

**Table 1:** Demographic and clinical characteristics of Medicare beneficiaries with BPH by race (January 1, 2010–December 31, 2018)

	All patients (*N*=31,699)	White (*N*=27,368)	BIPOC (*N*=4331)	ASD
Demographics*				
Geographical region of residence at index BPH diagnosis (%)				
South	12,877 (40.6%)	10,795 (39.4%)	2082 (48.1%)	0.024
Midwest	7018 (22.1%)	6368 (23.3%)	650 (15.0%)	0.029
Northeast	5575 (17.6%)	4943 (18.1%)	632 (14.6%)	0.013
West	6229 (19.7%)	5262 (19.2%)	967 (22.3%)	0.013
Clinical characteristics				
Charlson comorbidity score 1-year pre-index BPH diagnosis				
0	12,523 (39.5%)	11,266 (41.2%)	1257 (29.0%)	0.035
1	7467 (23.6%)	6486 (23.7%)	981 (22.7%)	0.020
2+	11,709 (36.9%)	9616 (35.1%)	2093 (48.3%)	0.038
Patients with other comorbidities 1-year pre-index BPH diagnosis (%)				
Cardiovascular disease	8028 (25.3%)	6939 (25.4%)	1089 (25.1%)	0.020
Diabetes mellitus	6149 (19.4%)	4836 (17.7%)	1313 (30.3%)	0.044
Dyslipidemia	21,215 (66.9%)	18,447 (67.4%)	2768 (63.9%)	0.011
Erectile dysfunction	3659 (11.5%)	3103 (11.3%)	556 (12.8%)	0.060
Hypertension	22,485 (70.9%)	18,999 (69.4%)	3486 (80.5%)	0.033
Obesity	2671 (8.4%)	2312 (8.4%)	359 (8.3%)	0.000

*ASD* average standardized difference, *BIPOC* Black, Indigenous, People of Color, *BPH* benign prostatic hyperplasia

*Patient age not included in the study included only patients aged 66 years during their index years

### BPH Surgical Procedure Type and the Likelihood of BPH Surgery

A total of 1853 male Medicare beneficiaries underwent BPH surgery during the study period. Among those who had a BPH surgery, TURP was the most common surgery for both BIPOC and White patients (56.8% vs. 49.4%, respectively). When comparing procedure type, BIPOC patients were more likely to undergo TURP than White patients (*p*=0.052; Table 2).

**Table 2:** Type of BPH surgery received by Medicare beneficiaries with BPH by race

	All patients (*N*=31,699)	Patients by race		
		White (*N*=27 368)	BIPOC (*N*=4 331)	*p-*value
Patients receiving any BPH surgery (%)	1853 (5.8%)	1661 (6.1%)	192 (4.4%)	<0.0001*
Patients receiving TURP (%)	929 (50.1%)	820 (49.4%)	109 (56.8%)	0.052
Patients receiving less invasive surgery† (%)**	406 (21.9%)	367 (22.1%)	39 (20.3%)	0.320
Other more invasive surgery†† (%)**	518 (28.0%)	474 (28.5%)	44 (22.9%)	<0.0001*

*p-value is less than 0.05

**Surgeries were reported aggregately given that some individual surgery types had less than 11 patients. According to Medicare data privacy agreements, findings with fewer than 11 patient counts cannot be publicly reported

†Less invasive surgeries included laser coagulation, PUL, TUIP, TUMP, TUNA, and WVTT

††More invasive surgeries include HoLEP, open simple prostatectomy, and PVP/HoLAP

*HoLEP* holmium laser enucleation of the prostate, *PUL* prostatic urethral lift, *PVP/HoLAP* photoselective vaporization of the prostate/holmium laser ablation of the prostate, *TUIP* transurethral incision of the prostate, *TUMT* transurethral microwave thermotherapy, *TUNA* transurethral needle ablation, *TURP* transurethral resection of the prostrate, *WVTT* water vapor thermal therapy

**Fig. 2:**
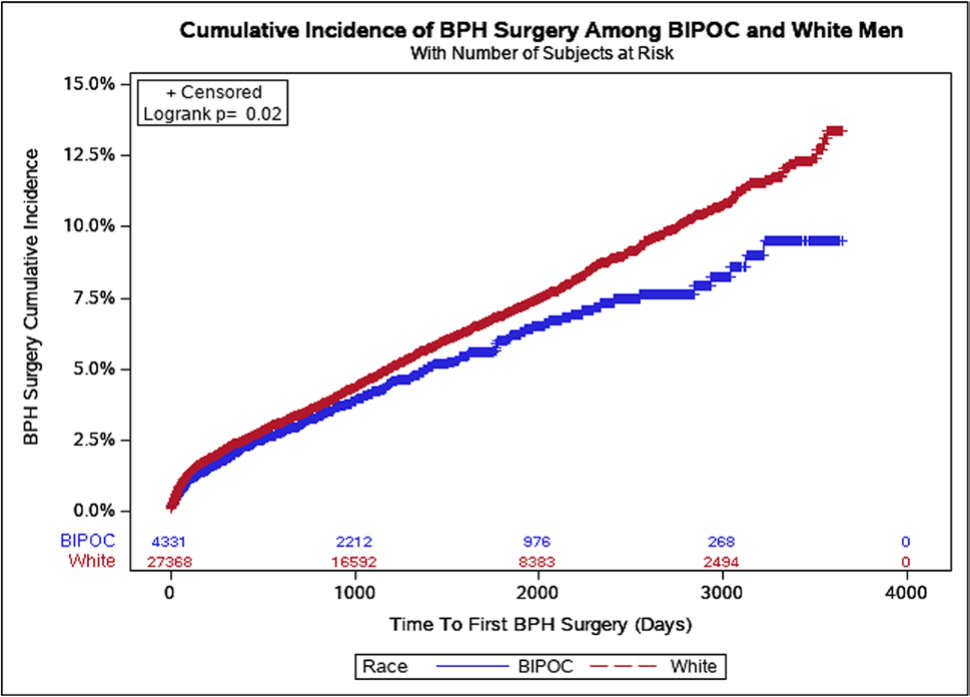
Time from benign prostatic hyperplasia (BPH) diagnosis to first BPH surgery in White vs. Black, Indigenous, and People of Color (BIPOC) men between 2010 and 2018

By the end of the study period, BIPOC men had significantly lower BPH surgery rates than White men (9.5% vs. 13.4%, respectively; *p*=0.02), as shown in Fig. 2. The multivariable-adjusted Cox regression model compared the likelihood of BPH surgery by race, controlling for patients’ geographical region of residence, Charlson comorbidity score, and baseline comorbidities, and found that BIPOC race was associated with a 19% lower likelihood of receiving BPH surgery than White race (HR, 0.81; 95% CI, 0.70–0.94; *p*=0.005) (Table 3).

**Table 3:** Patient characteristics associated with BPH surgery

Patient characteristics†	Hazard ratio (HR)	95% confidence interval (CI)		*p* value
BIPOC	0.81	0.70	0.94	0.005*
Geographical region at index BPH diagnosis				
Midwest	0.96	0.85	1.08	0.523
Northeast	0.75	0.65	0.87	<0.0001*
West	1.07	0.95	1.20	0.299
Charlson comorbidity score 1-year pre-index BPH diagnosis	1.01	0.98	1.04	0.627
Comorbidities 1-year pre-index BPH diagnosis				
Cardiovascular disease	1.04	0.92	1.18	0.507
Diabetes mellitus	0.96	0.83	1.10	0.557
Dyslipidemia	1.33	1.20	1.46	<0.0001*
Erectile dysfunction	0.94	0.82	1.07	0.341
Hypertension	0.99	0.89	1.10	0.869
Obesity	0.97	0.80	1.18	0.786

*p-value is less than 0.05

†Reference groups: White, South, no cardiovascular disease, no diabetes mellitus, no dyslipidemia, no erectile dysfunction, no hypertension, no obesity

*BIPOC* Black, Indigenous, People of Color, *BPH* benign prostatic hyperplasia

Male Medicare beneficiariess from the Northeast were significantly less likely to undergo BPH surgery compared to those from the South (*p*<0.0001); no other region was found to differ significantly in their likelihood of BPH surgery compared to the South. In addition, male Medicare beneficiaries with dyslipidemia were significantly more likely to receive BPH surgery than those without dyslipidemia (*p*<0.0001). No other comorbidity was associated with an increased likelihood of BPH surgery. Charlson comorbidity score was not significantly associated with a likelihood of BPH surgery.

#### Site of BPH Surgical Procedure by Race

**Fig. 3:**
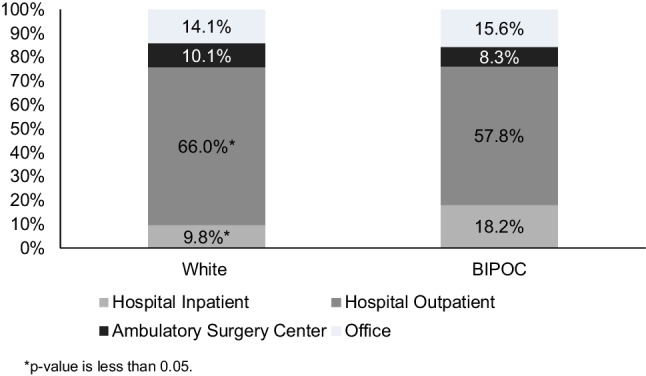
Site of BPH surgery by race. *p-value is less than 0.05

A significantly higher proportion of BIPOC men underwent BPH surgical procedures in the inpatient setting compared to White men (18.2% vs. 9.8%; *p*<0.001), while White men were more likely to receive surgical procedures in the outpatient setting compared to BIPOC men (66.0% vs. 57.8%; *p*=0.023; Fig. 3).

## Discussion

Racial and ethnic disparities in the care of patients in the US have been well documented. The Institute of Medicine concluded that even after controlling for insurance status, income, age, and severity of conditions, racial and ethnic disparities in the US healthcare exist. While many sources contribute to these disparities, bias, stereotyping, prejudice, and clinical uncertainty on the part of healthcare providers should be better evaluated and addressed to reduce inequities in healthcare [17, 18]. Disparities in urologic healthcare delivery and access have been researched and documented in the published medical literature [19]. However, this is the first study to demonstrate racial disparities in the rates of receiving BPH surgery and the site of service for BPH surgery among a cohort of Medicare beneficiaries. BIPOC men had lower BPH surgical procedure rates than White men and were more likely to undergo BPH surgical procedures in the inpatient setting. There was no significant association between the type of BPH surgical procedure and race. We believe that racial and ethnic disparities are the primary reason for the differences given that confounders including geographical region of residence, Charlson comorbidity score, and baseline comorbidities were controlled for.

These findings are consistent with those of recent studies evaluating treatment disparities in BPH. Antoine et al. identified BPH treatment disparities by race in a single-center review after adjusting for age, insurance status, major comorbidities, and type of pharmacotherapy [9]. Furthermore, in an analysis of the California medical database, Gill et al. found that Black patients were less likely than White patients to be treated with medications or surgery in the first year following their BPH diagnosis [8]. Given the heterogeneous National Medicare study population, this study adds to the evidence to support the generalizability of prior studies.

This study provides insight into the site of service of BPH surgical procedures, finding a higher proportion of BIPOC men who underwent procedures in inpatient settings compared to White men. Treatment site is an important consideration, given differences in both access and costs. With the increased use of less invasive technologies and the ubiquitous nature of ambulatory centers, outpatient facilities may provide easier access to care for patients relative to inpatient settings. However, it seems that BIPOC men are currently not receiving equal access to these less invasive and more convenient sites of care. Inpatient admission increases total costs to patients, especially when considering the indirect costs associated with care delivered in a more intensive setting. Further studies comparing the financial burden to payers and patients under these treatment scenarios are warranted.

Interestingly, patients in the Northeastern US were significantly less likely to undergo more invasive BPH procedure than those in other geographic regions, which may be attributed to the higher concentration of large academic medical centers in the Northeastern US. In addition, study participants with dyslipidemia were significantly more likely to receive BPH surgery than those without dyslipidemia. No other comorbidity was associated with an increased likelihood of BPH surgery. This could be due to the significant association between triglyceride/high-density lipoprotein ratio and BPH [20]. However, this retrospective database study did not enable us to ascertain the reasons for the observed associations, thus, further research is needed to understand the treatment patterns by these covariates.

Some potential limitations must be considered when interpreting these study results. First, the administrative claims data used in this study do not include potentially important confounders that may have influenced the results, such as physician/patient treatment preferences, symptom severity, or medication adherence patterns. The increased prevalence of BPH in Black men is likely secondary to other confounding factors, such as socioeconomic and environmental [10, 21]. Second, median length of follow-up was longer for White men (3.6 years) than for BIPOC men (2.8 years), which may indicate a higher mortality rate among BIPOC men, causing them to be censored in the analysis. However, at least a quarter of all men in each group had follow-up periods longer than 5 years. Administrative claims data rely on coding accuracy; however, there may be coding errors or incomplete or missing codes that may lead to under- or over-reporting of diagnoses or treatments [22, 23]. Administrative data may also have recording bias secondary to financial incentives and temporal changes in billing codes [22, 23]. Also, the results of this retrospective database evaluation reflect US patients with Medicare and may not be generalizable to patients with other types of health insurance or to patients in other countries, where clinical practice and reimbursement structure, healthcare accessibility, and treatment accessibility may differ.

Nevertheless, this study also had several strengths. By using real-world data from a large sample of men with BPH, this study reflects treatment patterns in the US. With 9 years of recent data (January 1, 2010–December 31, 2018), these findings represent the most current patterns of treatment and care. Previous research into race and BPH treatment patterns used data more than a decade old from a single US state and therefore may not reflect current treatment patterns [8]. Finally, numerous studies have identified differences in prostate cancer treatments by race. These findings indicate that racial differences extend to BPH surgical procedures. With the current data, urologists, payers, and policymakers can work to address these treatment differences and expand access to outpatient BPH surgical procedures for all patients.

BPH constitutes a large but often hidden burden on society given its high prevalence and impact on quality of life. It is therefore important to ensure that BPH treatments are equally accessible to all patients. Among a cohort of Medicare beneficiaries with BPH, there were notable treatment disparities when considering race. BIPOC men had lower rates of BPH surgical treatment than White men with no difference in the type of procedure performed. When treated, BIPOC men were more likely than White men to undergo procedures in inpatient settings. We believe that racial and ethnic disparities are the primary reason for the differences given that confounders such as geographical region of residence, Charlson comorbidity score, and baseline comorbidities were controlled for. The data are inherently limited; however, the findings provide a clear signal for further study. Further research is warranted to confirm the underlying causes for these associations. By understanding what may be driving these treatment disparities, patient access to BPH surgical procedures, especially those procedures performed in outpatient settings, may be improved.

Portions of this research were presented as a podium presentation at the American Urology Association (AUA) Annual Meeting 2022. 

### Supplementary Information

Supplementary file1 (DOCX 13.6 KB)

## Data Availability

This study used administrative claims data from the Centers for Medicare and Medicaid Services. Due to data use agreements signed with the Centers for Medicare and Medicaid Services, the data cannot be provided externally. Other researchers can purchase the same dataset to carry out similar analyses.

## Abbreviations

ASD: average standardized difference
BIPOC: Black, Indigenous, and People of Color
BPH: benign prostatic hyperplasia
CPT: Current Procedural Terminology (CPT®)
HoLEP: holmium laser enucleation of the prostate
HR: hazard ratio
ICD-9-CM, ICD-10-CM: International Classification of Diseases, Ninth and Tenth Revisions, Clinical Modification
IQR: interquartile range
PUL: prostatic urethral lift
PVP/HoLAP: photoselective vaporization of the prostate/holmium laser ablation of the prostate
SAF: Standard Analytical Files
TUIP: transurethral incision of the prostate
TUMT: transurethral microwave thermotherapy
TUNA: transurethral needle ablation
WVTT: water vapor thermal therapy
