# Supplementary material for: Is Race Associated with the Surgical Treatment for Benign Prostatic Hyperplasia? An Analysis of 30,000 Medicare Lives
Source: J Racial Ethn Health Disparities. 2023 Apr 24;11(1):528–34. doi: 10.1007/s40615-023-01538-0 (PMC10781854; doi:10.1007/s40615-023-01538-0)
Supplement: Supplementary file 1 — (DOCX 13.6 KB) [file 40615_2023_1538_MOESM1_ESM.docx]

**Appendix 1. BPH-related diagnosis and procedure codes**

| BPH-Related Event | | Code Type | Codes |
| --- | --- | --- | --- |
| BPH Diagnoses | | ICD-9-CM  ICD-10-CM | 600.xx  N40.x, N42.83 |
| Prostate Cancer Diagnoses | | ICD-9-CM  ICD-10-CM | 185  C61 |
| Bladder Cancer Diagnoses | | ICD-9-CM  ICD-10-CM | 188.x, 198.1, 223.3, 233.7, 239.4, V10.51  C67.x, C79.11, C79.19, D09.0, D30.3, D49.4, Z85.51 |
| BPH Surgical Procedures | Less Invasive | CPT | Laser coagulation (52647), PUL (52441, 52442, C9739, C9740), TUIP, TUMT (53850), TUNA (53852), WVTT (53854) |
|  | More Invasive | CPT | HoLEP (52649), open simple prostatectomy (55801, 55821, 55831), PVP/HoLAP (52648), TURP (52601, 52630, 52640) |
